# Attention and Outcomes Across Learning Conditions in L2 Vocabulary Acquisition: Evidence from Eye-Tracking

**DOI:** 10.3390/jemr18030021

**Published:** 2025-06-13

**Authors:** Yiyang Yang, Hulin Ren

**Affiliations:** School of Foreign Studies, University of Science and Technology Beijing, Beijing 100083, China; d202210543@xs.ustb.edu.cn

**Keywords:** eye tracking, vocabulary learning, incidental learning, artificial words, data-driven learning

## Abstract

The role of attention has been shown to be essential in second language (L2) learning. However, the impact of different learning conditions on attention and learning outcomes remains underdeveloped, particularly through the application of eye-tracking technology. This study aims to evaluate the effect of intentional learning conditions (i.e., data-driven learning) on vocabulary learning and attentional allocations. Twenty-six intermediate English L2 learners participated in the study to learn the usage of four artificial attributive adjectives in noun phrases (NPs). Learning outcomes were analysed to assess the types of knowledge developed, shedding light on the role of attention and the conscious processing of word usage. Eye-tracking data, collected using Eyelink 1000 plus, investigated gaze patterns and the allocation of attentional sources when applying the learned usage of adjectives. The results indicate that fixation stability and regression movements significantly differ under the impact of intentional learning conditions. Post-test results also indicate a shift in attention from the target adjectives to the associated nouns. These findings underscore the critical role of attention and highlight the influence of learning conditions on L2 vocabulary learning, providing practical implications and empirical validation for L2 educators and researchers aiming to enhance vocabulary instruction through intentional learning strategies.

## 1. Introduction

The role of attention in second language (L2) learning is widely acknowledged. Among the factors affecting L2 learning outcomes, such as first language (L1) experiences, the learner’s prior L2 knowledge, learning conditions, teaching methods, and word learnability, limited L2 researchers have focused on the effects of learning conditions on learners’ attention and awareness, especially in vocabulary learning.

In the field of second language acquisition (SLA), learners’ awareness and attention in knowledge development are generally assessed through subjective measures, regarding self-reported or verbalised rules as indications of explicit attention. As summarised by [1], subjective measures, such as confidence ratings, verbal reports, and source attribution selections, are often used to evaluate the types of knowledge that learners acquire. The key to identifying knowledge types using these measures lies in determining whether learners can verbalise rules and/or if automatic processing occurs when applying the knowledge, both of which indicate whether learners pay attention to the target linguistic features and are consciously aware of learning or applying the knowledge. However, studies like [2] highlight discrepancies between subjective and objective measures on the presence of attention in L2 knowledge application. It is thus unclear to what degree that any offline measure, whether subjective or objective, necessarily reflects attention when applying knowledge. Therefore, a more sensitive and immediate measurement that could reflect learners’ attention to target linguistic features during L2 vocabulary learning and production is necessary.

This study would thus implement eye-tracking technology, aiming to investigate learners’ attention underlying L2 vocabulary learning, to explicitly reveal the allocation of attentional resources during knowledge development. In addition to revealing learners’ attentional allocations, the eye-tracking data also aim to facilitate predictions about the development of word usage knowledge, particularly by exploring the relationship between learners’ attentional allocation and their knowledge acquisition under intentional learning. Therefore, this study attempts to answer the following research questions:

RQ1. What are the implicit and explicit characteristics of knowledge across incidental and intentional learning conditions?
RQ2. How does the intentional learning treatment affect learners’ attentional allocations and learning outcomes?

To address research question 1, learners’ performance was analysed in terms of test accuracy and the type of knowledge developed (implicit vs. explicit) by comparing results from the Grammaticality Judgment Tasks (GJTs), confidence ratings, and source attribution responses across learning conditions. Learners’ eye movement data were collected throughout the experiment to explore changes in the allocation of attention after the transitions from incidental learning to intentional learning. The relationships between learners’ attention and learning outcomes are also investigated for research question 2 to reveal any predictive power of eye-tracking measures on learning outcomes.

### 1.1. Conditions and Outcomes of Vocabulary Learning

The conditions of vocabulary learning are often conceptualised within a dichotomy of incidental and intentional learning (e.g., [3,4]). Incidental learning refers to the acquisition of vocabulary through meaning-focused activities such as reading (e.g., [5]), listening (e.g., [6]), media viewing ([7,8]), and gaming (e.g., [9,10]), where the primary focus is not on learning words but rather on understanding content or learning other literal knowledge. Empirical evidence supports the effectiveness of incidental learning, demonstrating that learners can acquire vocabulary through exposure to written texts, audio passages, songs, and television programs.

In contrast, intentional vocabulary learning involves deliberate and focused efforts to acquire words, often through structured activities such as using flashcards, studying word lists, engaging in writing exercises, or completing blank-filling tasks. This intentional approach is widely regarded as more effective, leading to faster and more substantial vocabulary gains, as well as better retention and higher levels of mastery [4]. Early research shows that intentional learning activities contribute to greater vocabulary acquisition compared to incidental methods (e.g., [11]), while in more recent studies, the investigation of the efficacy of incidental vocabulary learning is more predominant. The classification of vocabulary learning activities into incidental and intentional categories is useful for understanding the relative effectiveness of different approaches. However, while intentional learning is generally considered more straightforwardly effective, the extent to which the intentional and incidental conditions contribute to vocabulary learning, as well as how and what kinds of knowledge the two conditions can arouse, warrants further investigation.

The majority of these studies investigating the impact of conditions focus on learning outcomes, that is, the knowledge acquired. Based on the status of consciousness of awareness, knowledge can be categorised into two categories, namely implicit knowledge and explicit knowledge. Bialystok [12] distinguished implicit and explicit knowledge based on its consciousness and representations. Explicit linguistic knowledge is the conscious awareness of language rules that learners are able to articulate, while implicit linguistic knowledge contains the intuitive information of the target language, which is automatic and can be used spontaneously in language tasks. This definition is further developed, and the most commonly used criterion for distinguishing the type of knowledge is consciousness. According to Ellis [13], explicit knowledge involves conscious awareness of linguistics norms with controlled processing, which can be articulated, while implicit knowledge possesses intuitive awareness of linguistic knowledge, which is automatically processed and unable to be articulated. Despite those theoretical definitions, the current offline measurements of the two types of knowledge, however, as pointed out by [1], are still not sensitive enough. Therefore, this study will address this gap by implementing both offline and online measures of awareness to provide more precise evidence of learners’ attention, thus revealing the relationships among learning conditions, awareness, and learning outcomes.

### 1.2. Awareness and Attention in L2 Vocabulary Learning

While learning outcomes have received wide attention in second language acquisition (SLA) research, understanding the cognitive processes underlying L2 learning is equally crucial for the development of SLA theories and implications. In particular, the role of awareness and attention in cognitive processing has been emphasised in language learning, especially when examining how bilinguals process and learn an L2. Awareness enables potential encoding of linguistic features in L2 input, which is an early stage of language learning [14]. Awareness as noticing is a surface-level phenomenon, involving conscious attention to the occurrence of a particular stimuli, which aligns with the Noticing Hypothesis and the concept of lower-level awareness [15]. Awareness as understanding entails deeper cognitive processing through which an individual is able to recognise underlying regularities of the input [16].

With this aspect, the understanding of particular regularities can be developed from noticing, leading to the acquisition of implicit and/or explicit knowledge. However, while consciousness plays a vital role in the above-mentioned processes, how learning conditions influence learners’ consciousness status of awareness and knowledge is still under-explored. It is commonly agreed that intentional learning conditions arouse conscious attention; thus, laboratory studies such as those of John Williams [17] and fellow colleagues (see [18,19,20]) have implemented artificial language systems under incidental learning conditions, aiming to investigate the consciousness status of attention under incidental learning conditions. In Williams’s [17] seminal study, most students reported no conscious awareness of the hidden regulations after initial training, but around half of them were able to verbalise the hidden regulations at the end of the experiment. Subsequent studies have reported similar conclusions with diverse findings, indicating the presence of either conscious or unconscious attention in incidental learning conditions. Therefore, the consciousness status of attention paid under incidental learning conditions remains inconclusive. Building on the work of Williams and subsequent studies, this experiment developed an alternative (semi-)artificial word system with analogous rules to explore an earlier stage of vocabulary learning, that is, the learning of attribute adjectives.

Moreover, beyond memory tasks that test whether learners remember the target sentences, subsequent studies implementing the same artificial language paradigm have included additional measures of awareness and knowledge tests. These include verbal reports, comprehension tasks, alternative forced-choice questions, subjective measures, and follow-up questionnaires/interviews. However, discrepancies in the consciousness status of awareness may occur when using these offline measures [1,2]. While subjective measures tend to reflect both explicit and implicit knowledge, objective measures primarily detect explicit knowledge, often neglecting the presence of implicit knowledge. Some researchers also argue that the inability to verbalise linguistic rules may not necessarily indicate the presence of implicit knowledge. Rather, participants may simply lack meta-linguistic terminology required to articulate the rules [21]. It is thus unclear to what degree that any measure, whether subjective or objective, necessarily reflects the consciousness status of awareness and types of knowledge acquired and applied by learners. Therefore, alongside offline measures, eye-tracking technology was also employed in this study to reflect learners’ real-time attention when learning target words.

### 1.3. Eye Tracking as a Measure of Attention in L2 Vocabulary Learning

Eye-tracking technology provides precise insights into learners’ attentional processes. This timely measure offers a fine-grained understanding of real-time visual engagement and cognitive processes, capturing to which and for how long learners fixate on specific visual stimuli. Research employing eye-tracking technology in SLA has shifted from solely focusing on reading comprehension (e.g., [22,23]) to investigating vocabulary learning in reading (e.g., [24,25,26,27,28]), video viewing (e.g., [29,30]), as well as other learning activities (e.g., [31,32,33,34]).

Most research implementing eye-tracking technology has focused on vocabulary learning in the context of passage reading, as differences between skilled and less-skilled readers are primarily reflected in fixations, saccade length, and regressions. In reading contexts, ref. [23] found that word familiarity influences processing time, with readers allocate more attention to low-frequency and unfamiliar words. Less advantaged learners tend to exhibit longer fixations, shorter saccades, and more regressions [22]. Thus, to minimise the potential influence of prior knowledge of the target words and language proficiency on eye movement data, the present study has controlled these variables by employing (semi-)artificial words as the target vocabulary and recruiting intermediate English learners. As emphasised in Section 2.1, learning in such conditions takes place incidentally, without explicit awareness of target linguistic features. One of the most influential studies employing eye tracking to investigate vocabulary acquisition under incidental learning conditions is [25], which examines the relationship between fixation duration and the incidental learning of German verbs, proposing that longer fixations on target words during reading increase the likelihood of word recognition.

Subsequent researchers, for instance, who explore the connections between learners’ attention and learning gains under incidental learning conditions have also found positive relationships between the total reading time spent on the target words and learning outcomes such as form and/or meaning recognition (e.g., [24,28,35]), as well as between fixation duration and learning outcomes (e.g., [25,26,30,36]). However, compared to research conducted under incidental learning conditions, there is a scarcity of studies exploring the relationships between eye-tracking measures and learning outcomes under intentional learning conditions. Although some studies have reported similar patterns under intentional conditions [29,37], under which learning occurs with explicit awareness towards target linguistic features, these studies have primarily focused on learning strategies or modalities of input within a single learning condition. As a result, the impact of transitions between learning conditions remains insufficiently explored, while the transitions between different conditions demonstrate natural language learning situations. Moreover, some contradicted results have been revealed under intentional vocabulary learning, proposing that the decrease in attention could lead to greater learning gains (e.g., [32,34]). The inconsistency highlights the need for further investigation on the effect of intentional L2 vocabulary learning conditions on learners’ attention and learning outcomes.

Among the studies focusing on the effect of learning conditions, there is a relative paucity of research using eye-tracking data to evaluate (semi-)artificial vocabulary learning processes and outcomes. Of the eye-tracking research that does focus on the development of (semi-)artificial vocabulary, the majority has still been conducted under incidental learning conditions, often investigating the effects of distinctive reading activities on vocabulary learning. Godfroid’s team [25] inserted artificial nouns in reading to explore the difference in learners’ attention on known real words and unknown artificial words. The results demonstrate that more attention, which is reflected by a longer fixation duration, is paid to the combination of unknown artificial words and known real words, followed by artificial words, but more attention could not predict word retention.

Pellicer-Sanchez [38] used non-word nouns in passage reading to control participants’ previous knowledge, with non-words serving as artificial words to investigate the relationships between learners’ reading behaviours of these artificial words and learning outcomes. The results show a positive association between total reading time and vocabulary recall performance. Similar findings were obtained by [35], who matched 20 non-words with real words. Focusing on the impact of repeated reading on learners’ attention and learning gains, the results reveal that artificial words with a lower exposure frequency exhibit a longer fixation duration and fixation counts, presenting a positive relationship between attention and learning outcomes with the effect of exposure frequency. Besides solely reading-only activities, ref. [27] investigated younger learners’ learning gains of artificial nouns through reading-only and reading-while-listening tasks, reporting a shorter fixation duration in the reading-while-listening group, with better performance. The present study, however, moves beyond the use of simple reading activities by adopting a Type II incidental learning design. Subjects are explicitly instructed to learn some of the stimuli intentionally, while additional stimuli are embedded in the input but not salient to the learners, thereby making the learning of those stimuli incidental [39].

While the above-mentioned studies focus on incidental learning conditions, the study conducted by [34] matched verbs with 18 artificial words, aiming to evaluate the effect of picture illustration under intentional definition learning. Their results show that mismatched pictures largely hinder the acquisition of meaning. Regarding learners’ attention, they found that the influence of mismatched picture illustrations on learners’ fixation durations would eventually decrease through multiple exposures, which is positively correlated with knowledge test performance. Also, ref. [31] created 24 artificial nouns for intentional words learning. Although attention is not the main focus of their study, their results reflect differences in attention paid between monolinguals and bilinguals in vocabulary learning. Unlike the other studies, ref. [33] implemented (semi-)artificial sentences by inserting Finnish words into English sentences to explore the effect of massed and spaced reading conditions in sentence reading. The results also highlight the role of attention (i.e., fixation durations) in vocabulary learning, particularly under spaced reading conditions. The above-mentioned studies on intentional learning have primarily relied on a limited set of eye-tracking measures, namely first-fixation durations, first-fixation counts, and the total reading time, to assess learners’ attention. Nonetheless, other potential eye-tracking variables, such as the fixation rate, regression in, and regression out, have received relatively less attention, despite their relevance to reflecting learners’ attention in knowledge development and application. While the previous literature has paid attention to the reading time and fixation duration in vocabulary learning, the relationships between a broader range of eye-tracking measures and learners’ attention, as well as their associations with learning outcomes, are still underdeveloped.

**Figure 1:**
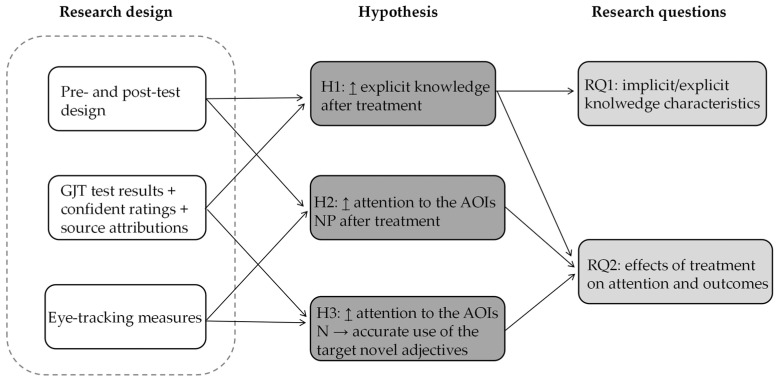
Conceptual diagram illustrating the relationships among research design, hypotheses, and research questions.

The majority of previous research on vocabulary learning has still relied on offline measures of attention, overlooking real-time indicators of attention in learning processes. Utilising eye-tracking technology to examine attentional processes in vocabulary learning, thus, draws our attention. In particular, we aim to examine learners’ attentional processes in word learning through sentences reading across different learning conditions. Therefore, to understand how learning conditions affect learners’ attention and learning outcomes, the aforementioned literature leads us to the following hypotheses, and Figure 1 presents the conceptual diagram based on research design, research questions, and hypotheses:

**Hypothesis 1 (H1).**  *After completing intentional treatment, learners will demonstrate significantly higher explicit knowledge gains of the target vocabulary.*

**Hypothesis 2 (H2).**  *Learners’ attentional resources, as indicated by eye-tracking measures, will be more intensively allocated to the target noun phrases (NPs) in the post-test compared to those in the pre-test, reflecting enhanced processing following intentional learning.*

**Hypothesis 3 (H3).**  *Learners’ attentional allocations, particularly the fixation duration, fixation rate, regression count, and total reading time to the associated nouns of the target NPs, will significantly predict the accuracy of word use in post-tests.*

## 2. Research Methods

This section outlines the research design, participants, materials, procedures, and data analysis methods employed to investigate the effects of intentional learning treatment on learning outcomes and learners’ attention, as stated in the research questions and hypotheses.

### 2.1. Participants

The study was conducted at a university in China, recruiting 30 undergraduate students who enrolled in non-English majors. The participants were recruited through a campus-wide call for volunteers, which included the distribution of printed posters in common areas of the campus and digital posters on social media. The posters provided a brief overview of the study, with the eligibility criteria (e.g., undergraduates, non-English majors, L1, English entry scores, etc.) and contact information. Students who expressed interest were subsequently screened to ensure they met the basic selection criteria before being invited. As an incentive, participants received CNY 30 (approximately USD 4) upon completing the experimental tasks.

All recruited participants are ESL (learning English as an L2) learners and have no previous experience of learning the target (semi-)artificial adjectives. Before participating in the study, the participants were asked to complete the reading section of the TOEIC exam. According to [38], the reading part of TOEIC scores can be divided into four categories aligning with the Common European Framework of Reference (CEFR): A1 = 60, A2 = 115, B1 = 275, B2 = 385, and C1 = 455. Intermediate ESL learners, that is, learners who scored 275–454 (*N* = 26), were invited to take the subsequent study. Table 1 presents the demography of the participants.

### 2.2. Stimuli

Many existing studies using (semi-)artificial language to investigate learning outcomes under incidental learning conditions have implemented an artificial linguistic system based on Williams [17], who inserted four artificial determiners into English sentences. Building upon Williams’s design and subsequent studies (see also, e.g., [19,20]), this study developed an alternative (semi-)artificial system with analogous rules. Instead of determiners, which are typically acquired at a later stage of language learning, this study employed four (semi-)artificial attributive adjectives that follow similar usage patterns to those in Williams’s work. The four words were retrieved from the ARC Nonword Database (http://www.cogsci.mq.edu.au/research/resources/nwdb, accessed on 12 February 2024) with orthographically existing bodies and legal bigrams. We also ensured that the words were orthographically simple to reduce learners’ working memory load.

The four artificial attributive adjectives are *gid*, *ros*, *ull*, and *nem*, which are the synonyms of English word *old*, but encoding distinct usage patterns of both *distance* (near vs. far) and *animacy* (animate vs. inanimate). The adjectives *gid* and *ros* are used with nouns that refer to near objects, whereas *ull* and *nem* describe distant objects. Regarding animacy, *gid* and *ull* refer to animate entities (natural, living, or moving things), while *ros* and *nem* are used with inanimate objects (man-made, non-living, or stationary items). Following a Type II experimental design, participants in this study received explicit training on the near/far distinction of the four artificial adjectives during the training phase, while the animacy regularity remained undisclosed at this stage. Therefore, the distance distinction was the salient pattern for participants, whereas the animacy rule was the hidden pattern. We then compiled all sentences following the correct patterns into a corpus using AntConc (https://www.laurenceanthony.net/software/antconc/, accessed on 1 March 2025) for the intentional treatment phase.

The nouns used in the noun phrases (NPs) in the training and exposure phases were adapted from [17] and comprised 12 animate and 12 inanimate nouns, each of which presented in both singular and plural forms. Nouns that were semantically incompatible with the meaning of ‘old’ were replaced with more contextually appropriate alternatives. Table 2 lists the NPs used in this study during the first two phases. There were 24 items in total (12 singular and 12 plural). Each form of each noun only appeared with one adjective (e.g., *gid dog* and *gid dogs*). As highlighted by [19], this ensures that participants who demonstrated knowledge development would accurately develop form–meaning connections (e.g., that *gid* is used with animate nouns) rather than form–form associations between the two pairs of adjectives (e.g., that any noun that can take *gid* [near] can also take *ull* [far]).

### 2.3. Materials and Instruments

#### 2.3.1. The Eye Tracker

An SR Research head-stabilised Eyelink1000 plus eye tracker with a desktop mount was used in this study. The participants were invited to the laboratory to conduct the whole experiment. When they sat in front of the display screen and used a chin rest to prevent head movement, they were informed to take a comfortable position. Before the experiment, the procedure was explained without revealing the aim of the study. After addressing any questions about the procedure, the experiment began. The Eyelink1000 plus system recorded the movement of the participants’ right eye. Prior to the main experimental tasks, the participants’ ability to clearly read the texts on the instructional screen (with the same font size and style as the main interface) was verified before the practice run and eye-tracking calibration to ensure they had normal or corrected-to-normal vision. Data accuracy was then verified using a nine-point calibration, followed by a validation grid. During the experiment, a fixation point appeared between each trial to allow for drift checking, and recalibration was carried out if necessary. Participants were instructed to read the texts normally for comprehension. The texts in the experiment were displayed at a resolution of 1280 × 960 pixels in black, using 16-point Courier New font on a white background to ensure monospaced lettering [40]. All lines were double-spaced.

#### 2.3.2. Grammaticality Judgment Test (GJT)

The GJT contains two sets of testing items, with each set consisting of 24 testing items. The contexts of the testing items were adapted from [19], which were completely new sentence contexts, none of which had appeared in the training materials. The participants needed to decide whether a given combination was suitable or unsuitable in the tests. The same adjective–noun combinations are used in Set 1 and Set 2, but they differ in terms of forms. That is, if a given noun appeared in its singular form in Set 1, then the same noun appears in the plural in Set 2, and vice versa. The order of Set 1 and Set 2 taken was counterbalanced, making sure that half of the participants took Set 1 as the pre-test and another half took Set 2 as the pre-test. The remaining test set was taken as the post-test. To better suit the eye-tracking experiment, sentences of the GJT were dedicated to be consistent in length (GJT Set 1: *M* = 13.13, *SD* = 1.394; GJT Set 2: *M* = 13.21, *SD* = 1.499), presented in a single line on the display screen.

#### 2.3.3. Confidence Ratings and Source Attributions

Binary confidence ratings and the indication of source attributions were collected following each GJT item, designed based on [1,41]. The learners were asked to select how confident they were in each judgment (high confidence vs. low confidence) and to state the basis of judgement (guess, intuition, memory, and rule). By considering the problem of response bias (e.g., [42]), binary confidence ratings (i.e., high confidence vs. low confidence) were adopted to reduce the effect of participants’ own reporting criteria for the confidence level.

The source attributions of judgement were collected to deal with certain situations, such as when accuracy and confidence are highly correlated. The participants were instructed in Chinese to select guess only when they made their judgment by purely guessing; to select intuition when they made their judgment based on a gut feeling that the NPs in the test items were suitable but could not verbally explain why; to select memory when their choice was based on recalling a similar sentence (or part of it) that appeared in any previous phases; and to select rule when their decision was based on a rule that they had integrated and they could verbalise. When selecting confidence ratings and source attributions, the learners were instructed to select the first option that came to mind.

### 2.4. Design

#### 2.4.1. Training and Exposure: Explicit Rule Learning

The purpose of the training was to introduce the rule of distance of the four artificial attributive adjectives, serving as incidental learning conditions for the acquisition of the animacy rule. The participants were informed that they would be tested after the training on the distance rule and that they could complete the training at their own pace. They were allowed to retake each section of the training as many times as they wished, until their gap-filling responses were at a satisfying level.

In the training phase, the participants were first presented with a list of the four artificial words and their meanings in English and Chinese, together with phonetic alphabets of the words and sample sentences with picture illustrations. They were instructed that the four adjectives are the synonyms of the English word *old* but with different usage patterns: two of them are used for distant objects, and the other two describe near objects. Afterwards, they were taught about the distance rule.

The students then completed a two-alternative forced-choice test, asking them to read sentences containing the four adjectives and to judge, as quickly as possible, whether the adjective indicated distance (far) or proximity (near). A Chinese translation of each sentence was provided, ensuring that the students could comprehensively connect the distance representation of the adjectives with the sentence meaning. They were also instructed that there would be tests afterwards so that they would pay attention to the usage of the target words rather than rely only on the translation or context. They could respond by pressing the *A* key (for near) or *L* key (for far), located at the left and right ends of the keyboard, respectively, to ensure a clear distinction between the two choices. Four practice sentences were given before the tests. The practice was untimed, whereas the test trials were timed, and the sentences disappeared when time limit was reached. Following the meaning judgment tasks, they completed an exposure task by filling the missing adjective that matched the meaning of sentences, with the Chinese translation provided for each sentence. A total of 24 gap-filling items were completed at this phase, and answers were given after each response.

#### 2.4.2. Intentional Treatment: Hidden Rule Learning

The treatment task, identical to the training tasks, took place after the pre-test (i.e., test phase 1). The learning activities in the treatment phase were data-driven, requiring the learners to induce language patterns from the concordance lines that were retrieved from the corpus. The learning aim at this phase was to figure out the hidden patterns (i.e., the animacy rule) from the concordance lines. As the learners were aware that they would be tested on the animacy rule afterward and the animacy rule was the main product of the activity, this treatment is considered an intentional learning treatment. The treatment phase was untimed.

#### 2.4.3. Pre-/Post-Tests

As noted in Section 2.3.2, the pre- and post-tests used separate test sets, conducted before and after the treatment, respectively. In the testing phase, the learners were required to complete the GJT, rate the confidence level, and attribute the source of judgement. The participants firstly judged the appropriateness of an adjective associating with an animate or inanimate object, responding by pressing the *A* key (appropriate) or *L* key (inappropriate). They then rated their confidence levels by pressing the *A* key (low confident) or *L* key (high confident) again and selected the source attributions of judgement by pressing the *A* key (guess), *S* key (intuition), *K* key (memory), and *L* key (rule). Four practice sentences were given before the pre-test trials to familiarise the participants with the test format. No practice was provided in the post-test, as the test format remained the same.

### 2.5. Procedure

Before the recruitment of the formal experiment, a pilot study was conducted with three volunteer participants to ensure the clarity of the instruction and guidance on the instructional screens, as well as the flow and consistency of the main tasks. The instructional screens not only presented the research procedure, instruction, and key dos and don’ts but also included a practice trial to familiarise the participants with the experience of text reading on the eye-tracker screen and the key-press operations.

**Figure 2:**
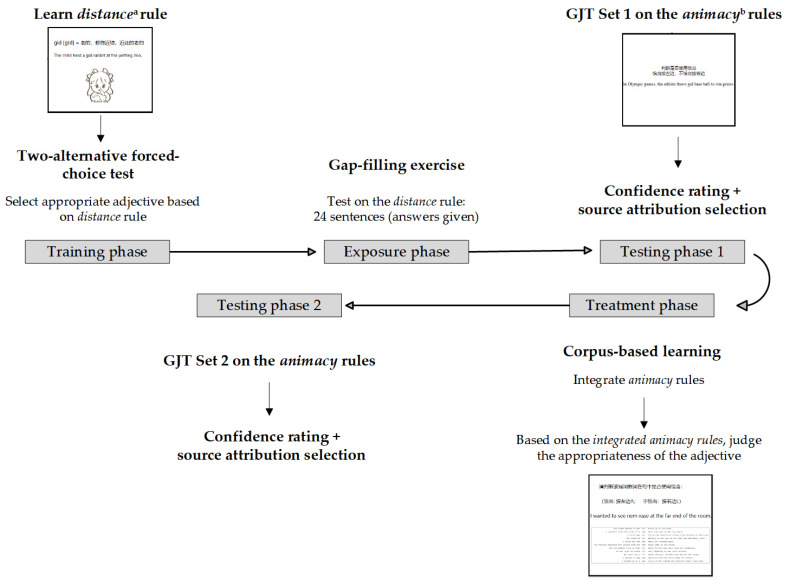
Research procedure of the study. *Note.* ^a^ = the explicit rule; ^b^ = the hidden rule, the learning of which during the training and exposure phases served as incidental learning. Prior to the formal trials in testing phase 1 (i.e., the pre-test), four practice items were provided.

In the formal experiment, the participants were exposed to five main phases, namely the training phase, exposure phase, testing phase 1, treatment phase, and testing phase 2. The procedure is illustrated in Figure 2. The five phases were conducted on eye-tracking screens, with eye movement data and participants’ responses recorded. While the time given for training tasks was unlimited, the subsequent tasks took around 15 min each. The participants were informed that during the experiment, they should keep their eyes focused on the screen, even when making a choice using the keyboard. After the post-test, the participants completed a brief questionnaire, collecting information about their age, gender, field of study, native language(s), and length of English learning.

### 2.6. Analysis

Behavioural and eye-tracking data were extracted using SR Research Data Viewer V2.4.1 by HR Research, Ottawa, ON, Canada (hereafter DV), a software for visualising and processing eye-movement data recorded with EyeLink eye trackers, and were analysed with models in R (http://www.R-project.org/, accessed on 3 June 2024). Area of interest (AOI) analysis was conducted to determine the parts that the participants drew attention to during the experiment. AOI analysis enables the selection of any area in a stimulus to reveal eye paths and movements. The AOIs were thus set to be the (semi-)artificial NPs in the sentences (e.g., *gid rabbit* and *ros desk*). Furthermore, considering that the animate–inanimate usage of the target adjectives depends on the living status of the associated nouns (e.g., *rabbit* vs. *desk*), the nouns in the NPs were marked as *N*, as subset AOIs.

Eye-movement data were first processed using DV. For each participant in each trial, fixations shorter than 80 ms were merged with neighbouring fixations that were located within 3 units or otherwise removed. Fixations longer than 800 ms were excluded. The overall data loss after cleaning was less than 1%. This pre-process aligns with standard practices in word-based eye-tracking research of psycholinguistics, where fixations shorter than 80 ms or 100 ms and longer than 800 ms are removed (see [38,40,43]). All eye-tracking variables in the Interest Area Output Report were generated and compiled into spreadsheets as the raw data for further analysis. Before statistical analysis, blank cells were manually inspected to determine whether the missing data were due to technical issues (e.g., eye tracker loss of signal) or participant behaviour (e.g., skipping an AOI). Behavioural-based missing values were filled with 0, while the trials with technical-based data loss was removed from analysis.

To answer research question 1, one-sample *t*-tests were conducted on the participants’ rates of accuracy from the post-test against chance-level performance (µ_0_ = 0.5) to examine whether there was statistical evidence provided for any learning effects in performance and if the performance was significantly above chance. The participants’ responses were categorised based on the selection of source attributions for each test item, in which the explicit category consisted of the source attributions rule and memory, and the implicit category included intuition and guess. For each knowledge category (implicit, explicit, and overall), paired-sample *t*-tests were used to compare performances across time (pre-test vs. post-test), with time as the within-subject factor and accuracy rate as the dependent variables.

To address research question 2, a series of statistical analyses were conducted to investigate how learners’ attention, captured through eye tracking, varies across conditions and could predict vocabulary learning outcomes. First, the learners’ responses in the pre- and post-tests were categorised into accurate and inaccurate items, with corresponding eye-movement variables retrieved from DV. Given the non-normal distribution of the eye-tracking data, non-parametric Wilcoxon signed-rank tests were used to analyse differences in the eye-tracking variables across time. By inputting time (pre-test vs. post-test) as the independent variable and all attentional measures as dependent variables, the analysis identified attentional variables that significantly differed depending on learning success across time. Next, the relationships between learners’ performance and variables with significant differences among groups were further evaluated by fitting logistic regression models. The participants’ accuracy (coded as 0/1) for each item in the post-test was used as the dependent variable, with relevant eye-tracking variables inputted as predictors.

Several potential threats to validity should be acknowledged in interpreting this analysis. First, while efforts were made to minimise noise in the eye-tracking data, there remains some possibility that the variability in eye behaviour or calibration drift may impact some fixation measures. Second, although the learners’ source attributions were used to categorise implicit and explicit knowledge, these attributions are self-reported and may not perfectly reflect the type of knowledge applied. Additionally, due to the relatively small sample size (*N* = 26), statistical power may be limited.

## 3. Results

This section presents the findings of the study based on the data collected through knowledge tests, subjective measures, and eye-tracking measures. The results are organised according to the types of data and their relevance to the research questions and hypotheses.

### 3.1. Numerical Data

Learners’ performance was firstly analysed across all participants, independent of time. The overall mean on the pre-test was 0.568, which was above the marginal line (0.5), indicating that learning of the hidden rule occurred under incidental exposure conditions; thus, subsequent analysis could be conducted with effective learning gains. Table 3 presents the accuracy for items based on subjective measures in the pre- and post-tests. The analyses of source attributions for each test item reported during the completion of the pre-test showed that the participants (across the four conditions) scored 0.61 (*SD* = 0.49) when basing their decisions on the explicit response categories (memory and rule knowledge) and 0.46 (SD = 0.50) when basing decisions on the implicit response categories (guessing and intuition). The mean accuracy was significantly above chance in explicit categories in the pre-test (*t* (776) = 6.14, *p* < 0.001). In the post-test, the mean accuracy was found to be significantly above chance in either condition (explicit categories: *t* (974) = 20.67, *p* < 0.001; implicit categories: *t* (112) = 2.14, *p* < 0.05).

**Figure 3:**
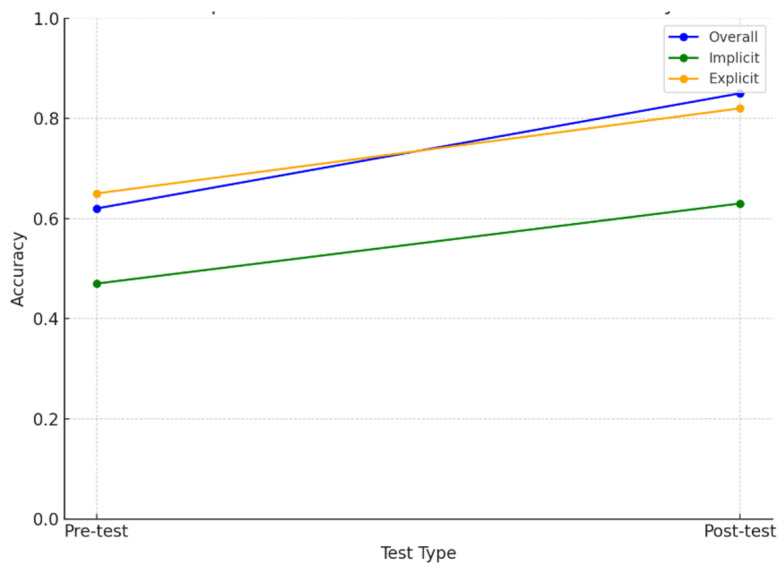
A comparison between pre-test and post-test accuracies.

Paired-sample *t*-tests were then conducted. Figure 3 presents the overall accuracy and the accuracy of each category across the two test conditions. Significant differences were found in the three pairs (overall: *t* (2156) = −9.76, *p* < 0.001; explicit categories: *t* (1750) = −7.73, *p* < 0.001; implicit categories: *t* (406) = −3.28, *p* = 0.0011), indicating an overall improvement in the accuracy. The conditional effect appears to be more pronounced for explicit categories, as evidenced by the larger *t*-value, whereas the improvement for implicit categories was still significant with a relatively smaller magnitude.

The numerical results on the rate of accuracy and types of knowledge demonstrate learning effects under both incidental (i.e., training phase) and intentional learning (treatment phase) conditions. However, greater learning gains were found under the intentional learning conditions in the acquisition of target hidden rule (i.e., the animacy constraint on NP pairing). Regarding the types of knowledge, the results of paired-sample *t*-tests showed that both implicit and explicit knowledge improved following the intentional treatment, with a greater gain in the explicit category, as reflected by a larger t-value, which could answer RQ1. This finding could also support H1 that intentional treatment would enhance the development of explicit knowledge more than implicit knowledge of the target attribute adjectives. The improvement in the overall accuracy from the pre- to post-test under the intentional learning condition, as shown by the paired-sample *t*-test results, could also provide evidence for RQ2. This demonstrates that the intentional learning condition does positively affect learning outcomes, leading to a higher rate of overall accuracy in applying the target animacy rule.

### 3.2. Eye-Tracking Data

In order to assess learners’ cognitive processes and attentional allocations when applying knowledge before and after the intentional learning treatment, fixations, regressions, and reading times of the AOIs were examined. Wilcoxon signed-rank tests were employed to assess statistically significant differences in eye-movement measures between pre-test and post-test conditions. Group effects were found for five variables: total fixation duration (TFD, the sum of all fixation durations on an AOI), total fixation rate (TFR, the proportion of all fixations on an AOI), second-pass fixation rate (SFR, the proportion of the second fixation on an AOI), total fixation count (TFC, number of fixations on an AOI), and regression in (RI, number of regressions to the AOI).

The five variables were further examined to explore the predictive power on GJT accuracy. For this, logistic regression models were fitted with the eye-movement variables as predictors of GJT results of the pre- and post-tests. The five subsets of variables were firstly analysed by regarding the target NPs as one single AOI. The output of the logistic regression models could not reject the null hypothesis when analysing the variables obtained in the pre-tests; however, the output suggested that the TFD, TFR, TFC, SFR, and RI are the predictors of performance on post-tests, as demonstrated in Table 4. The TFD, TFC, and RI were found to be significantly associated with post-test accuracy, with negative coefficients and relatively small effect sizes, suggesting that a longer fixation duration, greater number of fixations, and/or more frequent regressions into the target AOI result in lower accuracy. The TFR and SFR, however, exhibited a significant positive relationship with post-test accuracy, with relatively large effect sizes. These results suggest that an increase in the proportion of fixations and a higher rate of second fixations on the target NPs substantially improve test accuracy.

**Figure 4:**
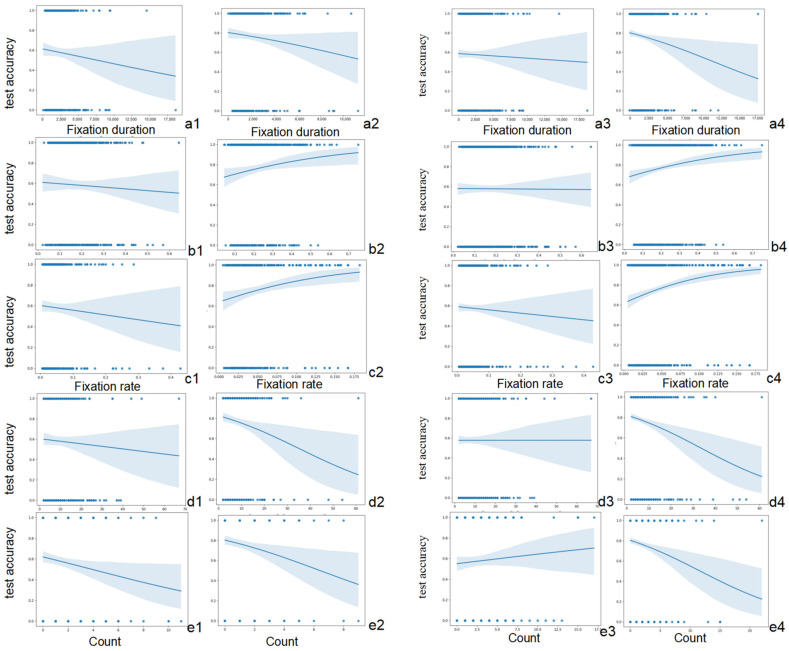
The relationships between participants’ eye-movement patterns and pre-test and post-test accuracies on AOI *N* (left) and AOI NPs (right). *Note.* Left column (for AOI N): relationships between TFD (**a1**,**a2**), TFR (**b1**,**b2**), SFR (**c1**,**c2**), TFC (**d1**,**d2**), and RI (**e1**,**e2**) measures and pre-test (**a1**,**b1**,**c1**,**d1**,**e1**), as well as post-test accuracy (**a2**,**b2**,**c2**,**d2**,**e2**). Right column (for AOI NPs): relationships between TFD (**a3**,**a4**), TFR (**b3**,**b4**), SFR (**c3**,**c4**), TFC (**d3**,**d4**), and RI (**e3**,**e4**) measures and pre-test (**a3**,**b3**,**c3**,**d3**,**e3**), as well as post-test accuracy (**a4**,**b4**,**c4**,**d4**,**e4**).

The five subsets of variables were further analysed, only analysing the associated nouns in the NPs as the interest area, named AOI *N*. The results also demonstrate that all five variables are not significant predictors of pre-test performance, while the other four variables, except TFD, obtained from AOI *N* in the post-tests are found to be significant predictors of post-test performance, as presented in Table 4. Figure 4 illustrates the relationships between participants’ eye-movement patterns and pre- and post-test accuracies. The *β* values were found to be distinct in different eye-tracking measures. Specifically, the TFC and RI exhibited negative associations with post-test accuracy, while positive relationships were found between post-test accuracy and the TFR as well as the SFR. Higher rates of fixations on AOIs may imply more frequent engagement with the target adjectives and associated nouns, and more attentional resources were allocated to the AOIs to integrate word usage patterns. Negative associations suggest that excessive fixations and regressions may be due to difficulty in processing the developed knowledge of the target NPs, leading to a decrease in the accuracy.

Among the significant predictors, the SFR and TFR, which exhibited positive associations, demonstrated the strongest and the second strongest predictive powers, respectively, as indicated by their effect sizes. A non-significant result was found for the TFD (*p* > 0.05) in the AOI *N*. This may indicate that the total time spent fixating on the target word alone does not significantly impact the accuracy. Rather, it is the proportions of fixations and second fixations that matter more. This aligns with prior research showing that the fixation duration alone is not always a reliable indicator of learning and comprehension, as it can be influenced by external factors such as reading strategies [44].

The investigation of changes in eye-tracking measures across incidental (i.e., training phase) and intentional (i.e., treatment phase) conditions addresses the second part of RQ2, which concerns whether intentional learning conditions could affect learners’ attentional allocations. The results show that more intensive attentional resources and a longer duration of attention were allocated to the target NPs, as reflected by the TFD, TFR, SFR, TFC, and RI. This finding confirms H2, suggesting that learners put more attentional resources to the target NPs in the post-test, indicating the involvement of greater cognitive load. However, H3 is only partially supported, as the results of the logistic regression model for the AOIs *N* (i.e., the associated nouns of the target NPs) show that the total fixation duration allocated to these AOIs did not significantly predict learning performance in the post-test. Nonetheless, other variables, that is, TFR, SFR, TFC, and RI, were found to significantly predict the accurate use of the target novel adjectives in the post-test.

## 4. Discussion

This section interprets the results in relation to the research questions, exploring the implications of the findings and providing possible explanations for the observed effects.

### 4.1. The Types of Knowledge Developed Under Incidental and Intentional Learning

In response to RQ1, the findings confirm that college L2 learners are able to learn the usage of (semi-)artificial attributive adjectives in a miniature linguistic system, as evident by the paired sample *t*-test results for overall performance. As for the effect of incidental learning (i.e., the training phase), the learning effect was found in the pre-test results, which is partially in line with previous studies investigating the effectiveness of incidental learning using (semi-)artificial languages (e.g., [19,20,45,46]). However, the one-sample *t*-test results showed that this learning effect could only be found in the explicit categories. This finding is consistent with previous studies that show that learners may employ conscious learning strategies under incidental learning conditions [47,48]. Additionally, the small learning effects could be explained by the low saliency of the target linguistic features during incidental exposure. In this study, four artificial words with novel usage patterns were embedded within the NPs of L2 sentences. Although the target NPs should have been noticeable to participants, as the meaning of the target adjectives and the distance–proximity rule were introduced at the training phase, the animate–inanimate pattern, as a hidden rule, might not have been salient enough for learners to encode. Therefore, they may not have had sufficient attentional resources to process and internalise the hidden usage pattern, even though the sentences contained only simple sentence structures and familiar vocabulary.

Regarding the outcomes of intentional exposure (i.e., the treatment phase), the improvement in test performance can be explained by the Noticing Hypothesis. Previous studies investigating the connection between attention and learning have also discussed the relationship between attention and intentional vocabulary learning (e.g., [29,37]). Based on the Noticing Hypothesis, learners should notice the target linguistic features from the input so that the information can be processed and converted into intake [49,50,51]. The intentional exposure thus makes the usage of the target NPs more salient, increasing the possibilities for learners to allocate their attention to the target linguistic features. In the training phase, the learners did not notice the hidden rule of the target words because the patterns were not salient enough to direct their attention to the target and convert input into intake. As noted previously, this could explain the small learning effect that occurred in the pre-test. The increase in saliency and attention after taking intentional learning could thus account for the improvement in post-test performance compared to pre-test results.

Moreover, a moderate learning effect was found in the post-test for both implicit and explicit categories. The relatively limited learning effects may be due to the influence of learners’ L1, Mandarin Chinese. Chinese does not include any classifiers that explicitly distinguish between animate and inanimate items. Rather, objects are grouped by characteristics like their shape, size, number, or granularity. This difference may affect learners’ ability to explore and internalise the use of words that possess novel usage patterns in the target linguistic system. While a learning effect was only found on the explicit categories of pre-test, the paired sample *t*-tests demonstrated a significant difference between pre-test and post-test performances. The findings indicate a presence of explicit (conscious) knowledge in the pre-test and both explicit and implicit (unconscious) knowledge in the post-test, as evidenced by the above-chance performance in both implicit categories (guessing or intuition) and explicit categories (memory or rule). This finding partially confirmed the observations in previous studies for the effect of incidental learning conditions on language learning (e.g., [45,46,52]), as well as under intentional learning conditions of language learning [30,32,33].

Nonetheless, the eye-tracking data indicated that reliance solely on subjective measures might not be sufficient to detect the presence of unconscious knowledge (i.e., implicit knowledge), as these tests lack the sensitivity required to evaluate cognitive processes underlying vocabulary learning. Subjective measures, such as confidence ratings and source attributions that were implemented in this study, and retrospective verbal reports primarily assess learners’ conscious awareness of acquired knowledge. However, these measures may fail to capture learning processes that occur with unconscious attention [1,42]. Both conscious and unconscious attentional processing can be reflected in eye-movement data, as revealed by fixations and regressions. Eye-tracking data thus offer more direct insights into the allocation of attentional resources during reading and vocabulary learning [53]. Given the advantages of eye-tracking technology, future research can be conducted to integrate eye-tracking technology with subjective measures to better distinguish different states of attention and levels of awareness, shedding light on both the processes and outcomes of L2 learning.

### 4.2. The Relationships Between Learners’ Attention and Learning Outcomes Across Incidental and Intentional Learning Conditions

Eye-movement measures were not found to be significant predictors of pre-test performance (i.e., after incidental learning), regardless of whether the AOIs were set as the target NPs or the nouns of the NPs. Given that learners were exposed to incidental learning conditions (i.e., before the pre-tests), where no hints or instructions were given to them on the hidden rule, visual engagement with the target NPs was likely to vary because of different cognitive strategies applied to process the input. Learning gains may also vary based on learners’ ability to encode the linguistic input. As a result, the variability in cognitive strategies and diverse ability to encode the usage patterns may have contributed to the lack of predictive power of eye-tracking data in the pre-test.

In contrast, eye-movement measures were found to be significant predictors of post-test (i.e., after intentional learning) outcomes, regardless of whether the AOIs were set as the target NPs or the nouns of the NPs. This finding suggests that intentional treatment guides learners’ visual attention in a selective manner, leading to deeper processing and higher chances of integrating linguistic input into memory representation. TFRs and SFRs on the target words were positively correlated with test performance, which means that the learners paid more attention to the target NPs at a global and late stage of processing, respectively, according to [40]. This finding might reflect learners’ ability to select and integrate useful information from the input during intentional learning. It can thus be deduced that intentional learning conditions can (re)direct learners’ attention towards the target linguistic features, stimulating the processing of usage pattern integration and, therefore, facilitating the initial construction of accurate form–meaning connection.

The results also revealed that while TFCs, Ris, and TRTs had significant predictive power, they negatively correlated with post-test performance. This finding contradicts previous findings that greater attention leads to better performance [35,38] but partially aligns with those of [27,34], who found that a shorter fixation duration may be associated with better performance. The phenomenon might be explained by the increase in cognitive load after engaging in more fixations and multiple times of regressions. As notified in the previous literature (e.g., [54]), eye-movement measures on fixations and regression could represent the increase in cognitive load, while learners regressed to the same locations searching for clues. Through this process, as their cognitive load increases, their judgment might be misled. The negative correlation between the RI and post-test performance may also suggest that less skilful learners may apply more frequent regressions. This finding aligns with [55] who find skilful learners present more frequent forward reading, while regression could be an indication of having difficulty in higher-level processing [56].

When examining the proportion of time that learners allocate attention to the target AOI against the total reading time, a different pattern emerges. In this study, TFRs and SFRs were positively related to test performance and possessed the greatest effect size. This demonstrates that successful learners were more likely to evaluate the important parts of the sentence. Thus, the percentage of time spent on the important parts, that is, the nouns of the NPs, was larger. Therefore, the successful learners spent a relatively greater proportion of time on AOIs than less successful learners. This result matches that of [57], who addressed the important and unimportant differences in fixations between important and unimportant text elements. Their finding suggests that the frequency of attentional allocation on important rather than unimportant text elements is associated with the amount of effort exerted to encode important information.

## 5. Conclusions

The present study explored the effects of learning conditions on learners’ attention and learning outcomes, while learners’ attention was assessed by eye-tracking measures. Small but significant learning effects were observed under incidental learning conditions, which aligns with the findings of [17] and subsequent studies (e.g., [20,27,38]). The results also demonstrated that intentional learning conditions promoted learning outcomes, especially in the development of implicit knowledge. This finding supports the claim that both implicit and explicit knowledge can be developed under either learning condition [1]. Eye-movement data showed medium and significant changes in learners’ attention paid to the target NPs, particularly to the nouns of the NPs after receiving intentional exposure. Notably, the learners’ fixation duration and reading time possessed lower statistical predictive power for learning outcomes than fixation rates and regression counts. This may imply that the allocation of attention might be more crucial than duration in vocabulary learning. Further research can be conducted to investigate the relationships between these eye-movement measures and learning outcomes.

The findings of this study can be generalised to learners with similar profiles. However, this generalisability is constrained by the demographics and educational context, as well as the sample size. For example, while the learning strategies and metalinguistic awareness might be different between English major and non-English major ESL learners, the allocations of attentional resources could differ if the target participants are set to be intermediate learners majoring in English. Therefore, the generalisability of the findings is limited by the specific population, task type, and the controlled lab setting of the experiment. Future research using larger, more diverse samples and different task designs is needed to strengthen external validity. In addition, as mentioned in Section 2.6, the potential impact of variability in eye behaviour or calibration drift, as well as the nature of knowledge type categorisation, should be acknowledged. Also, because of the relatively small sample size, the statistical power of the results may be limited. Another threat to the validity is that we did not test learners’ long-term word retention. Future studies investigating the effects of learning conditions on word retention can be considered to explore the relationships between learners’ cognitive strategies applied during word learning and long-term learning effects.

## Figures and Tables

**Table 1:** Demographic and background information of participants.

Background Survey Components		Frequency (*N*)	Percentage (%)
Gender	Female	16	61.5%
	Male	10	38.5%
Age	Range	18–23 (years old)	-
	Mean	21 (years old)	-
Major	Computer science and automation	8	30.8%
	Artificial intelligence	3	11.5%
	Energy and environment	3	11.5%
	Engineering	4	15.4%
	Other	8	30.8%
Year 1 and 2		19	73.1%
Year 3 and 4		7	26.9%
Years of English learning	Mean	13.3 (years)	-
Proficiency level	B1 level	8	30.8%
	B2 level	18	69.2%

**Table 2:** NP stimuli used in the training and exposure phases.

Non-Animate NPs		Animate NPs	
ros vase	nem book	gid dog	ull horse
ros phone	nem pen	gid goat	ull bear
ros sofa	nem table	gid bird	ull cat
ros desks	nem boats	gid rats	ull horses
ros cups	nem lamps	gid birds	ull cats
ros shelves	nem candles	gid monkeys	ull pigs

**Table 3:** One-sample *t*-test based on subjective measures in the pre-test and post-test. *Note*. * = *p* < 0.05, ** = *p* < 0.001.

Time	Condition	*N*	*M*	*SD*	*t*	*p*
Pre-test	Explicit knowledge	776	0.61	0.49	6.14	0.000 **
	Implicit knowledge	294	0.46	0.50	−1.28	0.200
Post-test	Explicit knowledge	974	0.78	0.42	20.67	0.000 **
	Implicit knowledge	112	0.64	0.48	3.14	0.0022 *

**Table 4:** Logistic regression model outputs of AOIs NP and N for post-test. *Note.* * = *p* < 0.05, ** = *p* < 0.001.

	Predictors	*β*	SE	Wald	*p*
AOI NP					
	TFD	−0.000	0.000	3.806	0.000 **
	TFR	2.777	0.708	15.881	0.000 **
	TFC	−0.036	0.011	9.885	0.000 **
	SFR	12.614	2.487	25.857	0.000 **
	RI	−0.102	0.034	9.295	0.003 *
AOI N					
	TFD	−0.001	0.000	1.965	0.160
	TFR	2.793	0.944	8.754	0.003 *
	TFC	−0.036	0.014	6.375	0.012 *
	SFR	9.454	2.773	11.527	0.000 **
	RI	−0.201	0.075	7.081	0.00 8*

## Data Availability

The datasets analyzed in this study are restricted by USTB CLL Review Board due to the inclusion of potential identifiers and cannot be made publicly available. Requests for access to the data can be directed to the USTB CLL Review Board at USTB_FS@163.com.

## References

[1] Rebuschat P. (2013). Measuring Implicit and Explicit Knowledge in Second Language Research. Lang. Learn..

[2] Maie R., DeKeyser R.M. (2020). Conflicting evidence of explicit and implicit knowledge from objective and subjective measures. Stud. Second Lang. Acquis..

[3] Webb S., Yanagisawa A., Uchihara T. (2020). How Effective Are Intentional Vocabulary-Learning Activities? A Meta-Analysis. Mod. Lang. J..

[4] Schmitt N. (2008). Review Article: Instructed Second Language Vocabulary Learning. Lang. Teach. Res..

[5] Pellicer-Sánchez A., Schmitt N. (2010). Incidental Vocabulary Acquisition from an Authentic Novel: Do Things Fall Apart?. Read. Foreign Lang..

[6] Pavia N., Webb S., Faez F. (2025). Incidental Vocabulary Learning from Listening to L2 Songs. Stud. Second Lang. Acquis..

[7] Feng Y., Webb S. (2020). Learning Vocabulary through Reading, Listening, and Viewing: Which Mode of Input Is Most Effective?. Stud. Second Lang. Acquis..

[8] Rodgers M.P., Webb S. (2020). Incidental Vocabulary Learning through Viewing Television. ITL-Int. J. Appl. Linguist..

[9] Calvo-Ferrer J.R., Belda-Medina J. (2021). The Effect of Multiplayer Video Games on Incidental and Intentional L2 Vocabulary Learning: The Case of Among Us. Multimodal Technol. Interact..

[10] Soyoof A., Reynolds B.L., Shadiev R., Vazquez-Calvo B. (2024). A Mixed-Methods Study of the Incidental Acquisition of Foreign Language Vocabulary and Healthcare Knowledge through Serious Game Play. Comput. Assist. Lang. Learn..

[11] Laufer B. (2005). Focus on Form in Second Language Vocabulary Learning. Eurosla Yearb..

[12] Bialystok E. (1978). A Theoretical Model of Second Language Learning. Lang. Learn..

[13] Ellis R. (2005). Measuring Implicit and Explicit Knowledge of a Second Language: A Psychometric Study. Stud. Second Lang. Acquis..

[14] Leow R. (2018). Explicit Learning and Depth of Processing in the Instructed Setting: Theory, Research, and Practice. Stud. Engl. Educ..

[15] Tomlin R.S., Villa V. (1994). Attention in Cognitive Science and Second Language Acquisition. Stud. Second Lang. Acquis..

[16] Schacter D.L. (2014). On the Relation between Memory and Consciousness: Dissociable Interactions and Conscious Experience. Varieties of Memory and Consciousness.

[17] Williams J.N. (2005). Learning without Awareness. Stud. Second Lang. Acquis..

[18] Hama M., Leow R.P. (2010). Learning without awareness revisited: Extending Williams (2005). Stud. Second Lang. Acquis..

[19] Rebuschat P., Hamrick P., Sachs R., Riestenberg K., Ziegler N., Bergsleithner J., Frota S., Yoshioka J. (2013). Implicit and Explicit Knowledge of Form-Meaning Connections: Evidence from Subjective Measures of Awareness. Not. Second Lang. Acquis. Stud. Honor Richard Schmidt.

[20] Zhao C., Kormos J., Rebuschat P., Suzuki S. (2021). The Role of Modality and Awareness in Language Learning. Appl. Psycholinguist..

[21] Hamrick P., Sachs R. (2018). Establishing evidence of learning in experiments employing artificial linguistic systems. Stud. Second. Lang. Acquis..

[22] Rayner K., Slattery T.J., Bélanger N.N. (2010). Eye Movements, the Perceptual Span, and Reading Speed. Psychon. Bull. Rev..

[23] Williams R., Morris R. (2004). Eye Movements, Word Familiarity, and Vocabulary Acquisition. Eur. J. Cogn. Psychol..

[24] Godfroid A., Ahn J., Choi I., Ballard L., Cui Y., Johnston S., Lee S., Sarkar A., Yoon H.-J. (2018). Incidental Vocabulary Learning in a Natural Reading Context: An Eye-Tracking Study. Biling. Lang. Cogn..

[25] Godfroid A., Boers F., Housen A. (2013). Gauging the Role of Attention in Incidental L2 Vocabulary Acquisition by Means of Eye-Tracking. Stud. Second Lang. Acquis..

[26] Wang Y., Gui M. (2024). Unveiling Cognitive Activities Associated with Longer Reading Times on Unknown Words in L2 Reading: An Eye-Tracking Case Study. System.

[27] Valentini A., Pye R.E., Houston-Price C., Ricketts J., Kirkby J.A. (2024). Online Processing Shows Advantages of Bimodal Listening-while-reading for Vocabulary Learning: An Eye-tracking Study. Read. Res. Q..

[28] Yi W., DeKeyser R. (2022). Incidental Learning of Semantically Transparent and Opaque Chinese Compounds from Reading: An Eye-Tracking Approach. System.

[29] Pi Z., Huang X., Wen Y., Wang Q., Zhao X., Li X. (2025). Happy Facial Expressions and Mouse Pointing Enhance EFL Vocabulary Learning from Instructional Videos. Br. J. Educ. Technol..

[30] Wang A., Pellicer-Sánchez A. (2022). Incidental Vocabulary Learning from Bilingual Subtitled Viewing: An Eye-tracking Study. Lang. Learn..

[31] Bartolotti J., Marian V. (2012). Language Learning and Control in Monolinguals and Bilinguals. Cogn. Sci..

[32] Bolger P., Zapata G. (2011). Semantic Categories and Context in L2 Vocabulary Learning. Lang. Learn..

[33] Koval N.G. (2019). Testing the Deficient Processing Account of the Spacing Effect in Second Language Vocabulary Learning: Evidence from Eye Tracking. Appl. Psycholinguist..

[34] Rop G., Van Wermeskerken M., De Nooijer J.A., Verkoeijen P.P.J.L., Van Gog T. (2018). Task Experience as a Boundary Condition for the Negative Effects of Irrelevant Information on Learning. Educ. Psychol. Rev..

[35] Mohamed A.A. (2018). Exposure Frequency in L2 Reading: An Eye-Movement Perspective of Incidental Vocabulary Learning. Stud. Second Lang. Acquis..

[36] Serrano R., Pellicer-Sánchez A. (2024). Online Processing and Vocabulary Learning in Massed versus Spaced Repeated Reading. Vigo Int. J. Appl. Linguist..

[37] Liu P.-L. (2014). Using Eye Tracking to Understand the Responses of Learners to Vocabulary Learning Strategy Instruction and Use. Comput. Assist. Lang. Learn..

[38] Pellicer-Sánchez A. (2016). Incidental L2 Vocabulary Acquisition from and While Reading: An Eye-Tracking Study. Stud. Second Lang. Acquis..

[39] Hulstijn J.H., Robinson P. (2001). Intentional and Incidental Second Language Vocabulary Learning: A Reappraisal of Elaboration, Rehearsal and Automaticity. Cognition and Second Language Instruction.

[40] Conklin K., Pellicer-Sánchez A., Carrol G. (2018). Eye-Tracking: A Guide for Applied Linguistics Research.

[41] Tannenbaum R.J., Wylie E.C. (2008). Linking English-Language Test Scores onto the Common European Framework of Reference: An Application of Standard-Setting Methodology. ETS Res. Rep. Ser..

[42] Dienes Z., Scott R. (2005). Measuring Unconscious Knowledge: Distinguishing Structural Knowledge and Judgment Knowledge. Psychol. Res. Psychol. Forsch..

[43] Cop U., Dirix N., Drieghe D., Duyck W. (2017). Presenting GECO: An eyetracking corpus of monolingual and bilingual sentence reading. Behav. Res. Methods.

[44] Rayner K. (2009). The 35th Sir Frederick Bartlett Lecture: Eye Movements and Attention in Reading, Scene Perception, and Visual Search. Q. J. Exp. Psychol..

[45] Rebuschat P., Hamrick P., Riestenberg K., Sachs R., Ziegler N. (2015). Triangulating measures of awareness: A Contribution to the Debate on Learning without Awareness. Stud. Second Lang. Acquis..

[46] Chen W., Guo X., Tang J., Zhu L., Yang Z., Dienes Z. (2011). Unconscious Structural Knowledge of Form–Meaning Connections. Conscious. Cogn..

[47] Leow R.P., Zamora C.C., Loewen S., Sato M. (2017). Intentional and Incidental L2 Learning. The Routledge Handbook of Instructed Second Language Acquisition.

[48] Tran R., Pashler H. (2017). Learning to Exploit a Hidden Predictor in Skill Acquisition: Tight Linkage to Conscious Awareness. PLoS ONE.

[49] Schmidt R., Robinson P. (2001). Attention. Cognition and Second Language Instruction.

[50] Schmidt R., Chan W.M., Chi S., Cin K.N., Istanto J., Nagami M., Sew J.W., Suthiwan T., Walker I. (2010). Attention, Awareness, and Individual Differences in Language Learning. Proceedings of the CLaSIC 2010, Singapore, 2–4 December 2010.

[51] Nelson R. (2011). Vigilance, Expectancy, and Noise: Attention in Second Language Lexical Learning and Memory. Second Lang. Res..

[52] Hamrick P., Rebuschat P., Rebuschat P., Williams J.N. (2011). How Implicit Is Statistical Learning?. Statistical Learning and Language Acquisition.

[53] Godfroid A. (2019). Eye Tracking in Second Language Acquisition and Bilingualism: A Research Synthesis and Methodological Guide.

[54] Zhai X., Dong Y., Wang S., Wang L., Yuan J. (2019). Exploring Eye-Tracking Analyses of EFL Learners’ Cognitive Processing of Reduced Relative Clause. Clust. Comput..

[55] Godfroid A., Loewen S., Jung S., Park J.-H., Gass S., Ellis R. (2015). Timed and Untimed Grammaticality Judgments Measure Distinct Types of Knowldge: Evidence from Eye-Movement Patterns. Stud. Second Lang. Acquis..

[56] Reichle E.D., Warren T., McConnell K. (2009). Using E-Z Reader to Model the Effects of Higher Level Language Processing on Eye Movements during Reading. Psychon. Bull. Rev..

[57] Kaakinen J.K., Hyönä J., Keenan J.M. (2002). Perspective Effects on Online Text Processing. Discourse Process..

